# Smaug destroys a huge treasure

**DOI:** 10.1186/gb4156

**Published:** 2014-01-27

**Authors:** Michael Götze, Elmar Wahle

**Affiliations:** 1 Martin Luther University Halle-Wittenberg, Institute of Biochemistry and Biotechnology, Kurt-Mothes-Strasse 3, Halle 06120, Germany

## Abstract

Smaug, a protein repressing translation and inducing mRNA decay, directly controls an unexpectedly large number of maternal mRNAs driving early *Drosophila* development.

See related research, http://genomebiology.com/2014/15/1/R4

## 

Regulation of translation and mRNA stability is a key aspect of early metazoan development. One of the best studied factors involved in these processes is the *Drosophila* protein Smaug. In this issue of *Genome Biology*, Chen *et al*. [1] report that a large number of maternal mRNAs in the fly embryo are probably regulated directly by Smaug.

## Maternal mRNA and its degradation in early development

During the first few cell divisions in animal development, the embryo’s genome remains silent. Development relies entirely on maternal RNA, a reservoir of RNA, including mRNA, copied from the mother’s genome during oogenesis and deposited in the developing oocyte, and protein synthesis is regulated exclusively at the levels of mRNA translation, stability and localization. In a process termed maternal-to-zygotic transition (MZT), control of development is then transferred to the zygotic genome. MZT obviously depends upon activation of the zygotic genome, but destruction of a large fraction of maternal RNA is also required. One reason seems to be that re-expression of specific genes from the zygotic genome can be limited to certain cells or regions of the embryo and thus, together with localization or localized destruction of maternal mRNA, contributes to differentiation. MZT ends with the first morphological alterations that depend on zygotic transcription.

In the development of *Drosophila*, the first 13 cycles of genome duplication and nuclear division are rapid and synchronous. As these nuclear divisions are not accompanied by cell divisions, a syncytium (or, according to a more meticulous definition, a plasmodium) results. With the beginning of the much slower 14th division cycle, approximately two and a half hours after fertilization, cell membranes form around the nuclei, which have arranged themselves as a monolayer at the periphery of the embryo. This transition from the syncytial to the cellular blastoderm marks the end of the MZT, while zygotic genome activation begins already around cell cycle 10. The maternal mRNA present before this stage represents about 55% of all protein-coding genes; that is, 6,000 or more transcripts [2-4]. Among them are mRNAs encoding housekeeping proteins, such as ribosomal proteins, but also RNAs coding for regulators of the cell cycle and proteins essential for patterning the embryo. Estimates of the fraction of maternal mRNAs degraded before cell cycle 14 range from 30% to 60% [2-4]. At least two degradation pathways with overlapping substrate specificities are involved. The maternal pathway is triggered by the activation of eggs caused by the process of egg-laying, but is independent of fertilization. Maternal decay - which might actually consist of several pathways sharing the characteristic of being independent of fertilization - starts immediately upon egg activation, but the degradation of specific maternal mRNAs may require the first 3 h of development. In contrast, the zygotic pathway occurs only in developing embryos; that is, this pathway depends on fertilization and zygotic transcription, and kicks in at about 2 h of development. Genetic evidence suggests that multiple factors contribute to the zygotic pathway, so the existence of more than one pathway is likely [3]. MicroRNAs are among the zygotic factors selecting maternal mRNAs for degradation [4,5].

## Smaug is essential for early development

One of the best-studied maternal mRNAs degraded through the maternal pathway is the *nanos* (*nos*) transcript, which encodes the protein directing the development of the posterior end of the embryo. The majority of the *nos* message is distributed uniformly throughout the embryo, remains translationally silent, and is degraded during the first 2.5 h of development. A small fraction of *nos* mRNA, however, which is localized in the germ plasm at the posterior end, escapes repression and destruction, providing the local source of Nanos protein that is essential for posterior patterning. Thus, degradation of maternal mRNA is not just mindless wholesale destruction, but a very finely tuned process that is interwoven with translational control and mRNA localization.

Among the regulators of *nos* mRNA is the protein Smaug, which binds the *nos* transcript through two Smaug recognition elements (SREs) residing in the 3’ UTR. In contrast to Bilbo Baggins’ eponymous adversary, *Drosophila* Smaug causes the destruction of the treasure it is sitting on, being responsible for both the translational repression and degradation of *nos* mRNA. Together with the piRNA machinery [6], Smaug recruits the CCR4-NOT complex to induce deadenylation as the first step in mRNA decay [7,8] and additional factors to repress translation [9]. Synthesis of Smaug starts after fertilization; the protein reaches peak levels at the syncytial blastoderm stage (cycles 10 to 13) and declines strongly during cycle 14. In accordance with this expression pattern, the development of Smaug-deficient embryos proceeds normally until division cycle 10, but further cycles are disturbed and cellularization never takes place. Importantly, zygotic genome activation is also impaired in *smaug* (*smg*) mutants [10]. As a deregulation of *nos* manifests itself later in development, the early phenotype of *smg* mutants indicates that there must be other important targets. The only other Smaug-regulated mRNA that has been studied in detail, *Hsp83* RNA (which is destabilized rather than translationally repressed by Smaug) [7], is unlikely to provide an explanation for the early embryonic defects of *smg* mutants.

## Smaug’s treasure is enormous

So how many and what kind of mRNAs are part of Smaug’s treasure? Microarray analyses have shown that at least 20% of all maternal mRNAs are substrates of the maternal degradation pathway [2,4]. Surprisingly, Smaug is required for the degradation of two-thirds of those, a minimum of 700, and presumably more than 1,000, mRNAs [2]. In their study, Chen *et al*. [1] examined the mRNAs translationally repressed by Smaug. For this purpose, they isolated polysome-associated mRNA from *smg* and wild-type control embryos and analyzed them by microarrays. The experiments resulted in a high-confidence set of 342 mRNAs that were more strongly polysome-associated in *smg* mutants, implying their Smaug-dependent repression in the wild-type. Using a statistical analysis, the authors extrapolated that as many as 3,000 transcripts, about one-half of the total number of mRNAs detectable in the early embryo, may be under translational control by Smaug. However, the two well-known Smaug targets, *Hsp83* and *nos*, were not among them. This was not unexpected: *Hsp83* RNA is destabilized but not repressed by Smaug, and *nos* mRNA has been reported (and was confirmed in this study) to be associated with polysomes, even though translation products are not detectable. This is an important caveat, showing that the presence of an mRNA in polysomal fractions does not exclude regulation by Smaug.

How many of the mRNAs regulated by Smaug are direct targets? Using immunoprecipitation of the protein followed by microarray analysis of associated RNA (RIP-chip), Chen *et al.* identified transcripts of 339 genes that are bound by Smaug. By means of a recently developed computational method, they then scanned the Smaug-bound RNAs and the high-confidence set of 342 translationally repressed RNAs for the presence of potential SREs, stem-loop structures with the loop sequence CNGGN_0-4_. Both in the bound and in the regulated RNAs, SREs were predicted with a 10-fold higher probability than in non-bound and non-regulated RNAs, respectively. In addition, the selected RNAs contained variant SRE sequences with probabilities matching the binding specificity of Smaug determined in earlier biochemical experiments: high-affinity sites were more enriched than low-affinity sites. These results come as no surprise with regard to the Smaug-bound RNAs; they merely support the reliability of their identification. However, a similar degree of enrichment of the SREs in the translationally repressed RNAs suggests that a large fraction at least of the high-confidence RNAs are direct targets of Smaug. By analyzing data from one of their previous studies [2], the authors also found SREs to be strongly enriched in mRNAs degraded in a Smaug-dependent manner, again suggesting a direct role for Smaug. Performing pairwise comparisons of RNAs bound by Smaug, repressed by Smaug (directly or indirectly) and destabilized by Smaug (directly or indirectly), Chen *et al.* found high degrees of overlap: two-thirds of the Smaug-bound RNAs were also destabilized by the protein, and three-quarters of the binders were also translationally repressed. Similarly, the destabilized and repressed RNAs overlapped to a large extent.

What about those RNAs that are destabilized or repressed but were not identified as Smaug ligands? These could be regulated indirectly by Smaug or they could be false-negatives in the RIP-chip experiments. From a significant enrichment of SREs in these classes of RNAs, Chen *et al.* concluded that a large fraction of the regulated RNAs are in fact direct targets of Smaug that escaped detection by RIP-chip.

As the number of Smaug-regulated mRNAs is large, they code for proteins involved in many aspects of biology. Messenger RNAs localized to the posterior pole were prominent among the Smaug targets, as were those encoding proteins involved in the regulation of DNA replication and transcription. More unexpectedly, the list of targets predicts regulatory effects of Smaug on protein folding and proteasome-dependent protein degradation, lipid droplets and even basic energy metabolism. With regard to metabolism, the majority of glycolytic enzymes were identified as potential Smaug targets, and enzyme assays confirmed a modest increase in hexokinase and phosphofructokinase activity in *smg* mutants.

## Conclusion

In summary, an unexpectedly large number of mRNAs in the early *Drosophila* embryo seem to be regulated directly by Smaug. Destruction of the protein during cell cycle 14 is presumably necessary to prevent degradation of zygotic transcripts, as many are derived from the same genes as maternal mRNAs. Since Smaug is necessary for zygotic genome activation, including, for example, the synthesis of microRNAs required for the zygotic pathway(s) of maternal mRNA decay, many additional RNAs are controlled indirectly by Smaug. The new data also suggest that Smaug targets are typically both destabilized and translationally repressed. The poly(A) tail is a potent stimulator of translation, so recruitment of the CCR4-NOT deadenylase by Smaug might be sufficient to cause both destabilization and repression. However, in the case of *nos*, translational repression goes beyond deadenylation [9]. The mechanisms by which Smaug brings about deadenylation and translational repression remain to be explored in more detail. Being derived from high-throughput data, the current list of Smaug-regulated RNAs will undoubtedly contain some fraction of false-positives in addition to the true targets, and there will be false-negatives as well. Many targets will very likely be confirmed by more detailed experiments as the list is used as a starting point for studies of Smaug-regulated biological phenomena and their contribution to the development of the fly embryo.

## Abbreviations

MZT: Maternal-to-zygotic transition; RIP-chip: RNA-binding protein immunoprecipitation followed by microarray analysis; SRE: Smaug recognition element.

## Competing interests

The authors declare that they have no competing interests.

## References

[B1] Chen L Dumelie JG Li X Cheng MHK Yang Z Laver JD Siddiqui NU Westwood JT Morris Q Lipshitz HD Smibert CA Global regulation of mRNA translation and stability in the early *Drosophila* embryo by the Smaug RNA-binding protein Genome Biol 2014 15 R4 10.1186/gb-2014-15-1-r4 24393533 PMC4053848

[B2] Tadros W Goldman AL Babak T Menzies F Vardy L Orr-Weaver T Hughes TR Westwood JT Smibert CA Lipshitz HD SMAUG is a major regulator of maternal mRNA destabilization in *Drosophila* and its translation is activated by the PAN GU kinase Dev Cell 2007 12 143 155 10.1016/j.devcel.2006.10.005 17199047

[B3] De Renzis S Elemento O Tavazoie S Wieschaus EF Unmasking activation of the zygotic genome using chromosomal deletions in the *Drosophila* embryo PLoS Biol 2007 5 e117 10.1371/journal.pbio.0050117 17456005 PMC1854917

[B4] Thomsen S Anders S Jang SC Huber W Alonso CR Genome-wide analysis of mRNA decay patterns during early *Drosophila* development Genome Biol 2010 11 R93 10.1186/gb-2010-11-9-r93 20858238 PMC2965385

[B5] Bushati N Stark A Brennecke J Cohen SM Temporal reciprocity of miRNAs and their targets during the maternal-to-zygotic transition in *Drosophila* Curr Biol 2008 18 501 506 10.1016/j.cub.2008.02.081 18394895

[B6] Rouget C Papin C Boureux A Meunier A-C Franco B Robine N Lai EC Pelisson A Simonelig M Maternal mRNA deadenylation and decay by the piRNA pathway in the early *Drosophila* embryo Nature 2010 467 1128 1132 10.1038/nature09465 20953170 PMC4505748

[B7] Semotok JL Cooperstock RL Pinder BD Vari HK Lipshitz HD Smibert CA Smaug recruits the CCR4/POP2/NOT deadenylase complex to trigger maternal transcript localization in the early *Drosophila* embryo Curr Biol 2005 15 284 294 10.1016/j.cub.2005.01.048 15723788

[B8] Zaessinger S Busseau I Simonelig M Oskar allows *nanos* mRNA translation in *Drosophila* embryos by preventing its deadenylation by Smaug/CCR4 Development 2006 133 4573 4583 10.1242/dev.02649 17050620

[B9] Jeske M Moritz B Anders A Wahle E Smaug assembles an ATP-dependent stable complex repressing *nanos* mRNA translation at multiple levels EMBO J 2011 30 90 103 10.1038/emboj.2010.283 21081899 PMC3020108

[B10] Benoit B He CH Zhang F Votruba SM Tadros W Westwood JT Smibert CA Lipshitz HD Theurkauf WE An essential role for the RNA-binding protein Smaug during the *Drosophila* maternal-to-zygotic transition Development 2009 136 923 932 10.1242/dev.031815 19234062 PMC2727558

